# Fetal circulation in left-sided congenital heart disease measured by cardiovascular magnetic resonance: a case–control study

**DOI:** 10.1186/1532-429X-15-65

**Published:** 2013-07-27

**Authors:** Bahiyah Al Nafisi, Joshua FP van Amerom, Jonathan Forsey, Edgar Jaeggi, Lars Grosse-Wortmann, Shi-Joon Yoo, Christopher K Macgowan, Mike Seed

**Affiliations:** 1 Department of Diagnostic Imaging, Hospital for Sick Children, University of Toronto, Toronto, Canada; 2 Department of Paediatrics, Division of Paediatric Cardiology, Hospital for Sick Children, University of Toronto, Toronto, Canada; 3 Departments of Medical Biophysics and Medical Imaging, Hospital for Sick Children, University of Toronto, Toronto, Canada

## Abstract

**Background:**

The distribution of blood flow in fetuses with congenital heart disease (CHD) is likely to influence fetal growth, organ development, and postnatal outcome, but has previously been difficult to study. We present the first measurements of the distribution of the fetal circulation in left-sided CHD made using phase contrast cardiac magnetic resonance (CMR).

**Methods:**

Twenty-two fetuses with suspected left-sided CHD and twelve normal controls underwent fetal CMR and echocardiography at a mean of 35 weeks gestation (range 30–39 weeks).

**Results:**

Fetuses with left-sided CHD had a mean combined ventricular output 19% lower than normal controls (*p* < 0.01). In fetuses with left-sided CHD with pulmonary venous obstruction, pulmonary blood flow was significantly lower than in those with left-sided CHD without pulmonary venous obstruction (*p* < 0.01). All three fetuses with pulmonary venous obstruction had pulmonary lymphangectasia by fetal CMR and postnatal histology. Fetuses with small but apex forming left ventricles with left ventricular outflow tract or aortic arch obstruction had reduced ascending aortic and foramen ovale flow compared with normals (*p* < 0.01). Fetuses with left-sided CHD had more variable superior vena caval flows than normal controls (*p* < 0.05). Six fetuses with CHD had brain weights at or below the 5^th^ centile for gestational age, while none of the fetuses in the normal control group had brain weights below the 25^th^ centile.

**Conclusions:**

Measurement of the distribution of the fetal circulation in late gestation left-sided CHD is feasible with CMR. We demonstrated links between fetal blood flow distribution and postnatal course, and examined the relationship between fetal hemodynamics and lung and brain development. CMR enhances our understanding of pathophysiology of the fetal circulation and, with more experience, may help with the planning of perinatal management and fetal counselling.

## Background

Left-sided congenital heart disease (CHD) includes a spectrum of abnormalities of the left ventricular inflow, ventricle, outflow tract, and aortic arch [1]. Left-sided CHD is accurately diagnosed with fetal echocardiography, which aids parental counselling, preparation for delivery, and postnatal management [2]. Selected mothers are now increasingly being offered fetal interventions in an attempt to modify the course of severe aortic stenosis and hypoplastic left heart syndrome (HLHS) with an intact or highly restrictive atrial septum (I/HRAS) [3].

Although ultrasound provides a wealth of physiologic information in these fetuses, the assessment of blood flow, one of the key parameters of hemodynamics, is technically difficult by ultrasound. Inaccuracies arise from problems obtaining an adequate angle of insonation, vessel area measurement, and assessment of variations in blood flow velocity across the vessel lumen [4,5]. In recent years phase contrast cardiovascular magnetic resonance (PC CMR) has gained importance as a clinical tool for blood flow quantification in postnatal patients with cardiovascular disease [6]. Cardiovascular magnetic resonance (CMR) is a widely available and safe technique for imaging the fetus and we have recently validated a PC CMR technique with metric optimised gating for use in late gestation fetal subjects [7,8].

Measurement of the distribution of fetal blood flow is potentially of interest to those caring for fetuses with CHD as it may provide new information about the impact of fetal hemodynamics on growth and development. For example, reduced flow through the left heart has been suggested as a cause for the progression of left-sided valve stenosis to HLHS [9], while abnormal cerebral perfusion has been implicated in the delayed brain development seen in fetuses with CHD [10]. In the setting of pulmonary venous obstruction (PVO), abnormal fetal cardiovascular physiology results in fetal pulmonary vascular disease causing severe hypoxia in the newborn, which may require emergency neonatal intervention [11]. Fetal MR also allows measurement of other physiologic variables that might be affected by abnormal hemodynamics such as cell metabolism, fetal brain size, and lung architecture [12-14]. The aim of this study was to evaluate the impact of various forms of left-sided CHD on blood flow using PC CMR. We also aimed to investigate the relationship between fetal hemodynamics and postnatal outcome and lung and brain development.

## Methods

This manuscript describes a single centre prospective cross-sectional case control study conducted between 2010 and 2012.

### Study population

Twenty-two pregnant women were recruited to undergo a fetal CMR after possible left-sided CHD was detected by fetal echocardiography at our institution. During the same study period, twelve normal controls were studied with the same technique at a similar range of gestational age. The results of this control group have been reported in our previous paper [8]. The fetuses were all studied during the third trimester with a median gestational age of 35 weeks and range of 30–39 weeks. Three subjects underwent fetal intervention to decompress pulmonary venous obstruction with CMR performed before and after the interventional procedure. The Hospital for Sick Children research ethics board approved the research protocol, and the expectant mothers gave written consent for CMR.

The fetuses were divided into four groups based on anatomical and physiologic type:

Group 1 Normal (n = 12): late gestation healthy controls.

Group 2 Aortic stenosis (AS) and/or aortic coarctation (CoA) (n = 7): fetuses with a small but apex forming LVs and aortic stenosis and/or a hypoplastic aortic arch or aortic coarctation with or without mitral stenosis (MS) but without PVO.

Group 3 Hypoplastic left heart syndrome (HLHS) with unrestrictive atrial septum (UAS) (n = 12): fetuses with HLHS with aortic atresia (AA) or severe AS with an UAS.

Group 4 HLHS with intact or highly restrictive atrial septum (I/HRAS) (n = 3).

There were no genetic syndromes identified in our cohort. One subject with coarctation had an Arnold Chiari malformation.

### CMR protocol

We performed the studies using a 1.5 T CMR system (Siemens Avanto, Erlangen, Germany). The CMR studies were performed on the same day as the last of three prenatal echocardiograms. Blood flow in the major fetal vessels was measured using PC CMR with metric optimized gating according to our previously published technique [8]. Measurements were made in the main pulmonary artery (MPA), ductus arteriosus (DA), superior vena cava (SVC), descending aorta (DAo) and umbilical vein (UV) [8]. Where possible, the ascending aorta (AAo) and right and left pulmonary arteries (RPA & LPA) were interrogated. A stack of T2 weighted single shot fast spin echo with half Fourier acquisition (HASTE) slices were prescribed axial to the fetal thorax and head to image the lungs and brain (slice thickness 5 mm, matrix size 320 × 256, field of view 350 mm, 50% phase oversampling, parallel imaging factor of 2, 1 average, echo time 145 ms, repetition time 1250 ms, scan time 1.3 s per slice). The entire examination was generally completed in 45 minutes, and the maximum time allowed for any study was one hour.

### CMR data analysis

Two radiologists (MS, BAN) post-processed every flow measurement using commercial software (Q-flow, Medis, Netherlands). Flows were indexed to the fetal weight according to our previously published technique [8]. The fetal weight was estimated from the fetal volume using a conversion factor based on fetal density developed by Baker *et al.*: 0.12 + 1.031 × fetal volume (ml) = MR weight (g) [15]. We used the same volumetry technique to estimate fetal brain volumes, and converted these to brain weights assuming a brain specific gravity of 1.04, based on a previously published conversion [16].

The combined ventricular output (CVO) was estimated as the sum of the MPA and AAo flows, accounting for an estimate of coronary blood flow of 3% of CVO, based on previous lamb data [17]. In fetuses with aortic atresia, the coronary blood flow is derived from the MPA flow via retrograde flow in the aortic arch. Flow in each vessel was expressed as net flow in ml/min/kg body weight. We were not able to measure the AAo flow in subjects with AA or severe AS due to the small size of the aorta. In seven fetuses with CHD and four normal fetuses we were not able to measure pulmonary blood flow (PBF) directly because of small vessel size and fetal motion. In those subjects, PBF was calculated from the difference between MPA and DA flow. Foramen ovale (FO) flow was estimated as the difference between the left ventricular output and PBF.

The HASTE imaging was reviewed to assess the lungs for the possibility of pulmonary lymphangectasia (PL). Visualization of high signal branching linear structures extending to the surface of the lung was required to make the radiologic diagnosis [14,18]. Lung histologic examination was performed after lung biopsy or during autopsy in all subjects with suspected PL.

### Echocardiography

According to the usual clinical protocol at our institution, an extensive prenatal and postnatal echocardiogram was performed in all subjects by one of five attending cardiologists. This included two-dimensional anatomical and hemodynamic assessment with colour flow and Doppler imaging. The diagnosis was generally made at around 20 weeks gestation, with follow up echocardiography at roughly 28 and 35 weeks gestation. The postnatal echocardiogram was performed within 24 hours of birth in all cases. Fetal cardiac anatomical assessment included a full sequential segmental analysis. The sizes of left-sided cardiac structures were measured and the presence of obstruction of pulmonary venous drainage, left ventricular inflow and outflow, and aortic arch obstruction and valve morphology were documented. The direction of blood flow in the aortic arch, the presence of an aortic coarctation, and any associated restriction of antegrade or retrograde aortic isthmus flow were investigated. Atrial septal restriction was assessed with 2D imaging and with colour and Doppler, including the ratio of antegrade to retrograde pulmonary vein velocity time integral (PV VTI), with a VTI ratio of less than five regarded as being consistent with PVO [19]. An assessment of cerebral vascular tone was performed with middle cerebral artery Doppler [20]. The possibility of placental insufficiency was investigated with umbilical artery (UA) Doppler and fetal biometry [21]. Postnatal echocardiography was performed according the guidelines published by the American Society of Echocardiography [22].

### Clinical outcome

The clinical outcome of each fetus with left-sided CHD was recorded, including birth weight and gestational age at birth, postnatal oxygen saturation range, surgical approach used, and length of time on the intensive care unit. Death or serious morbidity was noted with a variable follow up period from 1 to 24 months of age.

### Statistical analysis

Blood flows are expressed in ml/min/kg body weight. A two-tailed, unpaired *t*-test with unequal variance was used to compare flows between groups and Levene’s test was used to assess equality of variances. A model of the distribution of blood flows, as a percentage of CVO, was extrapolated from measured flows based on a constrained nonlinear optimization satisfying conservation of flow throughout the fetal circulatory system. Pearson’s correlation was used to investigate relationships with non-PC CMR measures. Statistical analyses were performed in MATLAB (Mathworks, USA).

## Results

**Figure 1:**
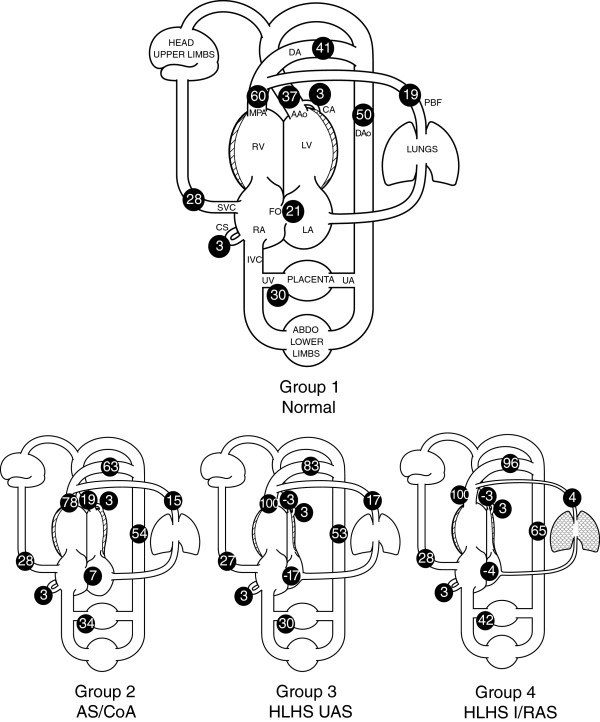
**Models of distribution of fetal blood flow as percentage of combined ventricular output by anatomical and physiological group, based on PC CMR measurements.** LA-left atrium; RA-right atrium; LV-left ventricle; RV-right ventricle; CVO-combined ventricular output; MPA-main pulmonary artery; AAo-ascending aorta; SVC-superior vena cava; PBF-pulmonary blood flow; FO-foramen ovale; DA-ductus arteriosus; DAo-descending aorta; UA-umbilical artery; UV-umbilical vein; CA-coronary artery; CS-coronary sinus; AS/CoA-aortic stenosis and/or aortic coarctation; PVO-pulmonary vein obstruction; HLHS-hypoplastic left heart syndrome; UAS-unrestrictive atrial septum; I/HRAS-intact or highly restrictive atrial septum.

Table 1 shows the demographic details of all subjects with suspected left-sided CHD. The mean and range of blood flows for each group are shown in Table 2. The demographic details and individual flows of the 12 subjects in the control group are given in our previous paper [8]. Figure 1 shows modelled flow distributions, as a percentage of CVO, for the entire fetal circulatory system, for each group.

**Table 1:** Subject demographics by anatomical and physiological group

**Diagnosis**	**Surgery**	**BW**	**GA**_**CMR**_	**GA**_**birth**_	**ICU**	**Outcome**
		**(kg)**	**(weeks)**	**(weeks)**	**(days)**	
**Group 1 Normal (n = 12)**						
Normal	No surgery	[2.0,4.0]	[30,39]	[37,41]	0	survived
**Group 2 AS/CoA (n = 7)**						
THAA	Hybrid, biventricular repair	2.9	37	37	68	survived
MS, AS	Ross and MV replacement	3.1	34	37	20	survived
CoA	Coarctation repair	3.2	34	38	2	survived
CoA	Coarctation repair	3.2	35	38	8	survived
MS, AS	Hybrid	3.6	38	38	6	survived
MS, THAA, VSD	Coarctation/VSD repair, resection supramitral ring	3.1	36	40	14	survived
CoA, Arnold-Chiari malformation	Coarctation repair	3.4	35	40	17	survived
**Group 3 HLHS UAS (n = 12)**						
HLHS UAS, MS, AA	Norwood	3.2	33	39	29	survived
HLHS UAS, MS, AA	no surgery	NA	34	NA	NA	termination of pregnancy (35 weeks GA)
HLHS UAS, MS, AA	Hybrid	3.6	34	40	18	survived
HLHS UAS, MA, AA	Norwood	3.1	35	39	17	survived
HLHS UAS, MA, AA	Hybrid	3.9	38	40	13	survived
HLHS UAS, MA, AA	Norwood	3.7	35	40	16	survived
HLHS UAS, MS, AA	Norwood	3.2	32	36	25	survived
HLHS UAS, MA, AA, LAI	Norwood	3.6	35	41	6	survived
HLHS UAS, MA, AA	Norwood	3.1	37	38	17	survived
HLHS UAS, MA, AA	Norwood	3.0	37	38	4	survived
HLHS UAS, MA, AA	Hybrid, Norwood	2.8	35	37	119	survived
HLHS UAS, MA, AA	Norwood	3.6	35	39	14	survived
**Group 4 HLHS I/HRAS (n = 3)**						
HLHS, MS, AA, TAPVC	Fetal stent, Norwood	3.1	32	38	99	survived
HLHS I/HRAS, MS, AA	Fetal stent, Hybrid	3.0	35	40	46	deceased (2 weeks)
HLHS I/HRAS, MA, AA	Fetal stent	NA	36	NA	NA	deceased (36 weeks GA)

Ranges are given as [min,max].

*BW* birth weight, *GA*_*CMR*_ gestational age at CMR, *GA*_*birth*_ gestational age at birth, *ICU* intensive care unit, *AS/CoA* aortic stenosis and/or aortic coarctation, *THAA* tubular hypoplasia of the aortic arch, *MS* mitral stenosis, *AS* aortic stenosis, *MS* mitral stenosis, *VSD* ventricular septal defect, *HLHS* hypoplastic left heart syndrome, *UAS* unrestrictive atrial septum, *AA* aortic atresia, *MA* mitral atresia, *LAI* left isomerism, *I/HRAS* intact or highly restrictive atrial septum, *TAPVC* totally anomalous pulmonary venous connection, *NA* data is not available or not appropriate.

**Table 2:** PC CMR measurements of blood flow by anatomical and physiological group

**CVO**	**MPA**	**AAo**	**SVC**	**PBF**	**FO**	**DA**	**DAo**	**UV**
**Group 1 Normal (n = 12)**								
540	327	198	147	106	107	220	273	160
[419,734]	[229,440]	[145,272]	[107,279]	[6,178]	[5,204]	[173,287]	[163,418]	[101,311]
**Group 2 AS/CoA (n = 7)**								
454	351	89	134	78	25	266	253	153
[392,569]	[321,400]	[51,152]	[107,152]	[40,152]	[−49,69]	[238,318]	[199,324]	[108,198]
**Group 3 HLHS UAS (n = 13)**								
456	456	0	132	81	−81	358	246	133
[350,596]	[350,596]	[0,0]	[70,231]	[30,140]	[−140,-30]	[300,456]	[165,337]	[101,165]
**Group 4 HLHS I/HRAS (n = 3)**								
331	331	0	112	21	−21	300	227	135
[114,479]	[114,479]	[0,0]	[21,195]	[10,38]	[−38,-10]	[89,427]	[75,307]	[57,251]

Flows are given as mean and range in ml/min/kg body weight. Ranges are given as [min,max].

*CVO* combined ventricular output, *MPA* main pulmonary artery, *AAo* ascending aorta, *SVC* superior vena cava, *PBF* pulmonary blood flow, *FO* foramen ovale, *DA* ductus arteriosus, *DAo* descending aorta, *UV* umbilical vein, *AS/CoA* aortic stenosis and/or aortic coarctation, *HLHS* hypoplastic left heart syndrome, *UAS* unrestrictive atrial septum, *I/HRAS* intact or highly restrictive atrial septum.

### Ventricular output and type of surgical repair

**Figure 2:**
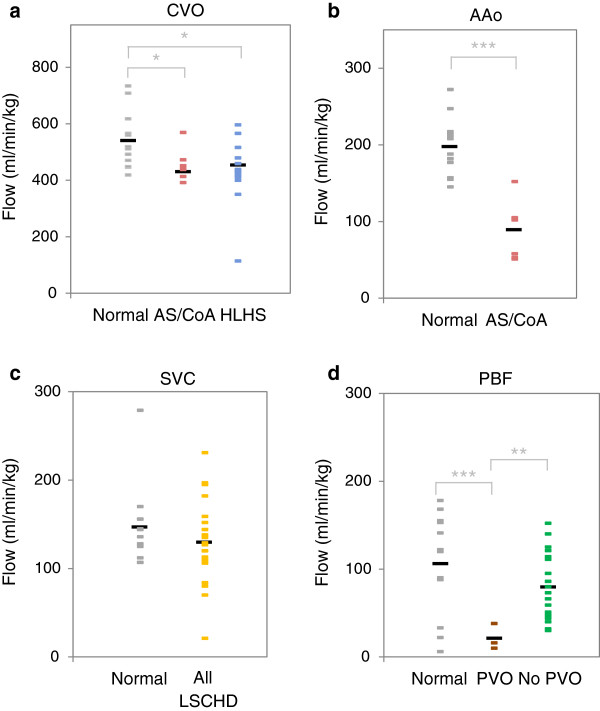
**Comparison of flows by anatomical and physiological group.** Mean (black bars) and individual measurements (coloured bars) for **(a)** CVO, **(b)** AAo, **(c)** SVC, and **(d)** PBF. Significance level is indicated (* *p* < 0.05, ** *p* < 0.01, *** *p* < 0.001). HLHS consists of Groups 3 (HLHS UAS) and 4 (HLHS I/HRAS). All Left-Sided CHD combines Groups 2 through 4. PVO consists of Group 4 (HLHS I/HRAS), while No PVO consists of Groups 2 (AS/CoA) and 3 (HLHS UAS). CVO-combined ventricular output; AAo-ascending aorta; SVC-superior vena cava; PBF-pulmonary blood flow; AS/CoA-aortic stenosis and/or aortic coarctation; PVO-pulmonary vein obstruction; HLHS-hypoplastic left heart syndrome; UAS-unrestrictive atrial septum; I/HRAS-intact or highly restrictive atrial septum.

Figure 2a shows the CVO for normal fetuses (Group 1), fetuses with HLHS (Groups 3 and 4), and fetuses with AS/CoA (Group 2). The mean CVO was 19% lower in fetuses with CHD than normals (*p* = 0.009), with no significant difference between fetuses with HLHS and AS/CoA. One fetus with HLHS with I/HRAS had a CVO of 114 ml/min/kg, dramatically lower than any other subject. This measurement was obtained 24 hours after atrial septal stenting, a procedure complicated by a myocardial infarction, which resulted in fetal demise the same day. One fetus with HLHS underwent late termination of pregnancy. All other fetuses with HLHS underwent single ventricle palliation, either with a Norwood procedure or Hybrid (stenting of the arterial duct and bilateral branch pulmonary artery banding) in the first days of life.

In our normal control group, the mean contributions of the right and left ventricles to the CVO were 327 ml/min/kg (60% of CVO) and 214 ml/min/kg (40% of CVO), respectively. In subjects with AS/CoA (Group 2), the mean relative contributions of the right and left ventricles were 351 ml/min/kg (78% of CVO) and 103 ml/min/kg (22% of CVO), respectively, with a significantly lower AAo flow (*p* < 0.001) compared to normals (Group 1). The reduction in AAo flow in this group was associated with reduced FO flow (*p* = 0.0013) compared to normal fetuses, as shown in Table 2 and Figure 1.

All fetuses with AS/CoA (Group 2) were eventually able to undergo a biventricular repair, although two underwent Hybrid procedures in the neonatal period with subsequent conversion to a biventricular repair at six months of age. One fetus underwent a neonatal Ross procedure and mitral valve replacement. The remainder of the fetuses underwent coarctation repairs, usually with some patching of the aortic arch. Fetuses with lower AAo flows tended to require more complex repairs, although there was a considerable overlap of AAo flows with respect to type of repair.

### Pulmonary blood flow, pulmonary venous Doppler, and lymphangectasia

Figure 2d demonstrates low PBF in the three fetuses with PVO (Group 4) compared with those with no PVO (Groups 2 and 3) (*p* = 0.0016). There was a wide range of PBF in fetuses with left-sided CHD without PVO (Groups 2 and 3), similar to normal fetuses (Group 1). We found no correlation between fetal PBF and postnatal ASD gradient or neonatal oxygen saturation in fetuses with HLHS with UAS (Group 3).

**Figure 3:**
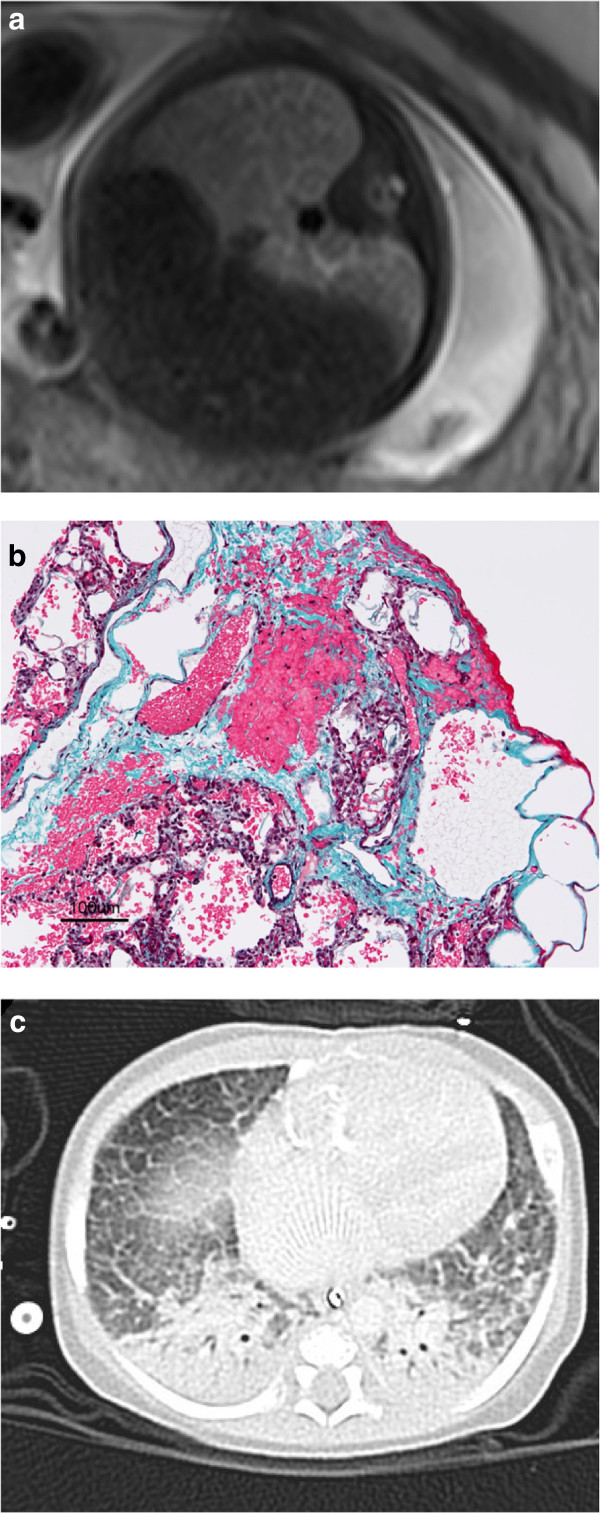
**Fetal lung CMR suggestive of pulmonary lymphangectasia confirmed by lung biopsy and postnatal CT in subject with pulmonary venous obstruction. (a)** Axial single shot fast spin echo with half Fourier image of the fetal chest demonstrating linear structures extending to the surface of the lung (arrow) suggestive of pulmonary lymphangectasia. The diagnosis was confirmed by **(b)** lung biopsy showing dilated lymphatics (arrow) and **(c)** postnatal high resolution CT showing thickening of the interlobular septae (arrow).

**Figure 4:**
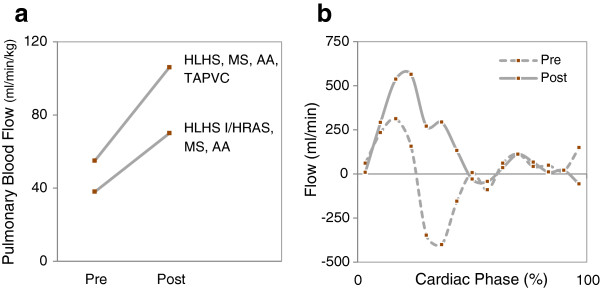
**Increased pulmonary blood flow following fetal atrial septal stenting. (a)** Pulmonary blood flow in the two cases that underwent fetal intervention to decompress the pulmonary veins, where a fetal CMR flow assessment was performed (pre) before and (post) one to two weeks after the intervention. **(b)** Flow curves from the left pulmonary artery in one subject that underwent fetal intervention to decompress the left atrium. The dotted line shows the flow (pre) prior to intervention and the solid line show the flow (post) one week after the intervention. HLHS-hypoplastic left heart syndrome; I/HRAS-intact or highly restrictive atrial septum.

All three fetuses with HLHS with I/HRAS had low PBF and PV VTI ratio less than 5 by Doppler. These were the only three fetuses who had lung MR appearances suggestive of PL. PL was confirmed by biopsy or autopsy in all three. The fetal lung imaging, postnatal chest CT, and lung biopsy of one subject with obstructed pulmonary venous return is shown in Figure 3. All three of these fetuses underwent fetal atrial septal stenting. In two of these, blood flows were measured by CMR shortly prior to the procedure and one week after. Figure 4 shows a considerable increase in PBF in these two fetuses following the interventions. Despite the improvement in PBF, all of the fetuses undergoing in-utero stenting had bad outcomes. There was one fetal demise resulting from thrombosis complicating the stent, which caused a myocardial infarction. One died soon after the Hybrid with a combination of pulmonary hypertension and low cardiac output syndrome. One survived a long intensive care course but sustained significant brain injuries.

**Figure 5:**
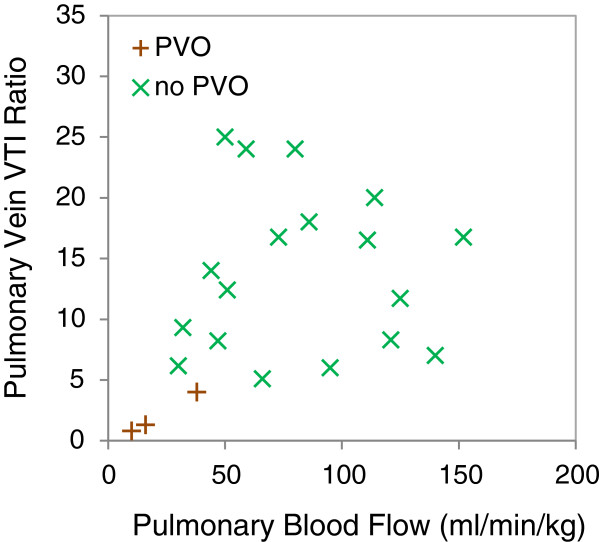
**Relationship between pulmonary vein velocity time integral ratio by Doppler and pulmonary blood flow by CMR.** Fetuses with pulmonary lymphangectasia (PVO, Group 4) are compared with fetuses with normal lungs (no PVO, Groups 2 and 3). VTI-velocity time integral; PVO-pulmonary vein obstruction.

All fetuses with PL by CMR had a PV VTI ratio of less than 5. Low PBF was also present in some fetuses without PVO, including normal fetuses. However, high PBF was associated with the absence of pulmonary venous obstruction and PL. The relationship between PBF, PV VTI, and PL is shown in Figure 5.

### Cerebral blood flow and brain volumetry

Cerebral blood flow could not be directly measured due to the small size of the vessels supplying and draining the brain. However, we used SVC flow as a surrogate for cerebral blood flow, assuming the flow from the other sources of venous return to the SVC, primarily the musculoskeletal system of the head and upper extremities was likely to be reasonably constant. In normal fetuses, there was a narrow range of SVC flows around the mean of 147 ml/min/kg with the exception of one outlier, which had high flows throughout the circulation likely due to a hyperdynamic state of unknown cause. Figure 2c shows a similar mean SVC flow of 130 ml/min/kg in all fetuses with left-sided CHD, but with higher variance (*p* = 0.018).

**Figure 6:**
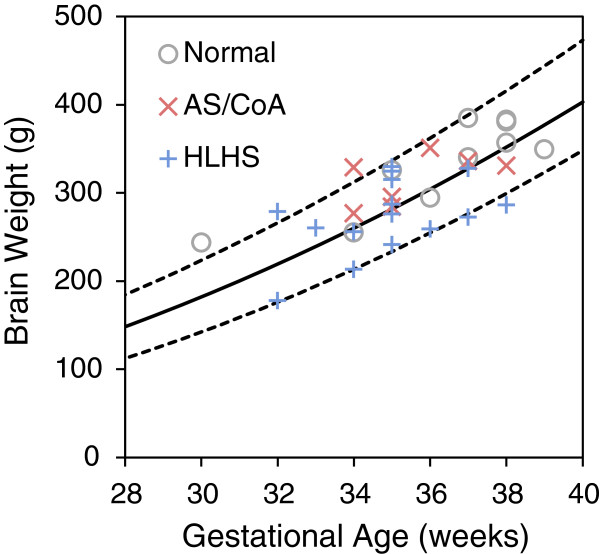
**Relationship between brain weight and gestational age.** Normal (Group 1), AS/CoA (Group 2), and HLHS (Groups 3 & 4) anatomical and physiological groups plotted against normal median (solid line) and 5th and 95th percentiles (dashed lines), based on a previously published autopsy series [24]. AS/CoA-aortic stenosis and/or aortic stenosis; HLHS-hypoplastic left heart syndrome.

MR derived brain weights for the normal and the disease groups were plotted on a graph of normal ranges taken from a previously published large autopsy series, shown in Figure 6[23]. Six of the fetuses with CHD had brain weights at or below the 5^th^ centile for gestational age, while none of the normal control fetuses had brain weights below the 25^th^ centile. These smaller brains were found in subjects with CHD despite normal fetal weights and normal birth weights.

We found no correlation between brain weight and flow in the SVC, AAo or CVO when the fetuses with left sided CHD were analysed according to anatomical subtype or as a whole. Neither did we find any correlation between SVC flow or brain weight with middle cerebral artery or UA Doppler resistive index, or aortic isthmus size or Doppler gradient.

## Discussion

The feasibility of measurement of the distribution of the fetal circulation using PC CMR in fetuses with left-sided CHD is established by this study. When CMR was performed towards the end of the pregnancy, a comprehensive dataset could be obtained in all subjects with a scan time of less than one hour.

The distribution of the normal fetal circulation has been studied in animals and humans using radioactive microspheres and ultrasound [17,24-28]. The CMR findings in normal controls are detailed in our previous paper [8] and are generally consistent with previous estimates. One new observation was the wide range of PBF and FO flow found in normal fetuses. In the setting of a rather constant AAo flow, we found an inverse relationship between these two competing sources of venous return to the left ventricle. There is some evidence to suggest this variation in FO flow results from variation in the streaming pattern of the umbilical venous return at the ductus venosus and FO and the associated changes in pulmonary vascular resistance that occur in response to changes in placental function [17,29]. Recent experimental lamb and human Blood Oxygen Dependent MR data showing that brain oxygenation is maintained at a constant level during fetal hypoxia and hyperoxia indicate that the primary function of this variation in streaming may be the maintenance of a constant oxygen content of the blood supplied to the fetal brain [30,31].

Cardiac output [32], pulmonary vascular resistance [33], and cerebral vascular resistance [34] have been measured in fetuses with HLHS using Doppler and are discussed below. The accurate identification of a restrictive atrial septum in HLHS by PV VTI ratio is an example of the clinical utility of fetal hemodynamic assessment in CHD, as this enables accurate identification of fetuses that may require emergency neonatal intervention [35,36]. We were interested in exploring whether flow measurements made in fetuses with left-sided CHD using CMR could contribute useful additional information to the usual echocardiographic assessment.

### Right and left ventricular output

In keeping with previous echocardiographic fetal flow measurements, our study indicates an average reduction of approximately 20% in CVO in HLHS compared to normals [32,36]. This would suggest that while the RV compensates to some degree for the absent LV output in HLHS, there may be an upper limit on RV performance. In general, the 20% reduction in CVO in HLHS and other less severe forms of left sided CHD appears to be well tolerated, although it may explain the mild growth restriction seen in newborns with HLHS [37]. The very low CVO seen in the fetus that suffered a myocardial infarction following atrial septal stenting is an example of how the measurement of flow by CMR may be helpful for assessing ventricular dysfunction, as the severity of the cardiac insufficiency had not been appreciated by echocardiography in this case. With the exception of this fetus, we noted an increase in MPA flow in all fetuses with left-sided CHD compared with controls. This increase in RV output is expected because the increased afterload imposed by left ventricular outflow tract obstruction results in reduced FO flow and therefore increased RV preload. The increase in RV output is also associated with increased DA flow, which balances the absence of a contribution to DAo flow from the aortic isthmus. It also compensates for the reduction in AAo flow by providing retrograde flow to the aortic arch. Retrograde aortic isthmus flow is the principle supply of head and upper extremity flow in fetuses with HLHS, but also contributes and average of one quarter of head and upper extremity flow in fetuses with less severe forms of left-sided CHD such as those with AS and CoA. The demonstration of retrograde isthmus flow in fetuses that subsequently develop coarcation is in keeping with previous fetal echocardiographic studies [38], and suggests that in some patients with arch hypoplasia the discrete isthmus narrowing may occur only after birth, possibly in conjunction with postnatal ductal constriction.

In fetuses with HLHS the upper body, lower body, and pulmonary circulation share a common supply of blood flow from the MPA. Rudolph speculated that this would result in a higher than normal oxygen content of blood reaching the pulmonary circulation, and a lower than normal oxygen content of blood reaching the cerebral circulation, with the possibility of cerebral and pulmonary vasodilation [17]. However, he also suggested that anatomic obstruction at the atrial septum and aortic isthmus might limit pulmonary and cerebral blood flow. We observed similar mean flows to each of the vascular beds in HLHS compared with normal controls, although as Rudolph predicted, we found wide variation in these flows as follows.

### Fetal pulmonary circulation

As streaming of the umbilical venous return across the ductus venosus towards the FO is likely to be less effective in the fetal circulation with left-sided obstructive CHD, and third trimester pulmonary vascular resistance is inversely proportional to the oxygen content of pulmonary arterial blood [17], the range of PBF in fetuses with left-sided CHD may reflect variation in oxygen delivery from the placenta. Cordocentesis data from normal late gestation pregnancies suggest that umbilical vein saturation ranges from 60 to 80% [39]. We would therefore suggest that in fetuses with left-sided CHD with good placental function without PVO we might expect high PBF. We did indeed find examples of fetuses with left-sided CHD with PBF approaching 200 ml/min/kg, similar to the most pulmonary vasodilated normals, and at 81 ml/min/kg (18% of CVO), the mean PBF in the HLHS with UAS group was similar to the 106 ml/min/kg (19% of CVO) found in normals. The low PBF seen in fetuses with PVO is likely due to the increased lung resistance resulting from pulmonary venous hypertension. By contrast with the postnatal circulation, the presence of the DA in the fetal circulation offers an alternate route for the RV output to avoid the pulmonary circulation. A relatively small increase in lung resistance could therefore result in the dramatic fall in PBF that is seen in these fetuses.

Previous authors have estimated a prevalence of restriction at the atrial septum in HLHS of up to 22%, with an intact atrial septum present in 6% [40]. The elevated pulmonary venous pressure in fetuses with HLHS with I/HRAS not only results in low PBF but also in developmental and histological changes within the lung vasculature, characterized by arterialization of pulmonary veins and thickening and reduced calibre of the small pulmonary arteries [41]. This is frequently associated with dilation of the pulmonary lymphatics, which is thought to be due to increased lymphatic flow resulting from increased hydrostatic forces in the vessels [42]. Antenatal detection of an I/HRAS is currently routinely achieved by assessment of the pulmonary venous flow pattern by Doppler echocardiography [19,35,42]. A low PV VTI ratio is highly suggestive of atrial septal restriction. It is therefore not surprising to find that fetuses with CMR findings suggestive of PL had low PV VTI ratios. These fetuses also had uniformly low PBF by CMR (Figure 5). However, there were examples of fetuses with similarly low PBF without evidence of PVO, presumably reflecting upstream pulmonary vasoconstriction. By contrast, no fetus with high PBF by CMR had signs of PL or an abnormal VTI, so normal or high PBF by CMR would appear to be predictive of an absence of PVO.

Restriction at the atrial septum is one of the most important risk factors for adverse outcome in fetuses with HLHS before and after surgery for both staged reconstruction and heart transplantation [43,44]. Affected neonates present with profound hypoxemia and may die or sustain a significant insult before medical or surgical intervention. A number of centres now perform intrauterine fetal left atrial decompression with the aim of preventing further pulmonary vascular damage and reversing some of the pathological changes already present [45]. The increase in PBF to near normal levels seen in the three fetuses undergoing fetal atrial stenting, shown in Figure 4, supports this approach. While the initial postnatal clinical presentation of these patients was more stable than those born at our centre with I/HRAS in the era prior to fetal intervention, their long-term outcome remains poor [46]. We would suggest that the antenatal diagnosis of PL by CMR complements the Doppler assessment of the fetal pulmonary circulation in HLHS with I/HRAS. CMR provided additional evidence of the presence of significant pulmonary vascular disease in these fetuses and the identification of PL supported the case for urgent neonatal or antenatal atrial septal intervention.

### Fetal cerebral circulation

Children with HLHS have reduced IQ and a variety of deficits of neurocognitive function [47]. While some of this is likely to be due to neurologic insults occurring during cardiac surgery and the post-operative period, recent studies have suggested that newborns with HLHS have brain abnormalities at birth [10] and that their brain growth and metabolism are abnormal towards the end of gestation [48]. One possible etiology is inadequate cerebral perfusion due to abnormal fetal hemodynamics. In the normal fetus, the brain is perfused by antegrade flow from the left ventricle via the AAo and aortic arch. The AAo is a relatively large vessel, carrying a mean of 37% of the CVO in the normal fetus [8]. In fetuses with any degree of left-sided CHD, antegrade flow in the AAo is limited, and the DA supplies a significant proportion of the cerebral blood flow via retrograde flow in the aortic arch. The reduced pulsatility found in the middle cerebral artery in fetuses with HLHS is one piece of evidence to suggest that cerebral vascular hemodynamics are affected by this anatomy [34,49].

We were interested in the relationship between fetal brain growth and cerebral perfusion. We found that fetuses with left-sided CHD had a mean SVC flow similar to normals (Figure 2c). However, by contrast with the normal controls that had a narrow range of SVC flows, we found wide variations in SVC flow from 21–231 ml/min/kg. Possible causes for low SVC flow include obstruction to retrograde flow at the aortic isthmus and stealing to other parts of the circulation in the setting of limited CVO. Fetal cerebral blood flow is also affected by the oxygen content of the blood in the aortic arch, with a powerful hypoxic cerebral vasodilation mechanism in operation [50]. Furthermore, the normal fetus appears to regulate the oxygen content of ascending aortic blood despite variation in oxygen delivery from the placenta though variation in the ductus venosus and foramen ovale shunt [8,29-31]. However, the fetus with left-sided cardiac obstruction is not likely to be able to vary foramen ovale flow to regulate the oxygen content of ascending aortic in the same way as the normal fetus due to the obligate left to right or reduced right to left shunt at atrial level. Therefore the oxygen content of ascending aortic blood and therefore the cerebral blood supply are likely to be more strongly coupled to oxygen delivery by the placenta. The wide variation in SVC flow we found in fetuses with left-sided CHD may reflect the need for more cerebral vasoreactivity to adequately oxygenate the brain in the setting of normal variations in placental oxygen delivery. The absence of any correlation between UA Doppler and SVC flow could be interpreted as contradictory to this hypothesis. However, UA Doppler has been shown to be an insensitive measure of late onset placental insufficiency [51]. The very low SVC flow of 21 ml/min/kg seen in the fetus with a myocardial infarction is in keeping with the observation that brain-sparing physiology fails with severe fetal distress, which may be a sign of imminent fetal demise [52].

We were surprised to find no relationship between middle cerebral artery Doppler and SVC flow. However, Rudolph has suggested that the middle cerebral artery Doppler is not an accurate reflection of cerebral vascular resistance in HLHS because of the possibility of upstream obstruction [17]. Another explanation for the lack of correlation we found between middle cerebral artery Doppler, SVC flow, and brain volume may be that our CMR study represented a “snapshot” of the fetal circulation during late gestation. Our finding of lower brain weights in subjects with CHD is in keeping with a previous study, which detected brain weights more than 2 standard deviations below the normal mean in 27% of fetuses with HLHS [53]. While the significance of abnormalities of fetal cerebral perfusion are not known with respect to long-term neuro-developmental outcome, we believe that an approach combining fetal brain imaging and PC CMR may contribute to the growing body of information being gathered regarding this issue.

### Study limitations

The small sample size of this study mandates a cautious approach to evaluating the findings. Our protocol examined a single time point during fetal life and it would have been interesting to know how the distribution of flow varied over time. The current technique is not suitable for making flow measurements at younger gestational ages, although this would be desirable. For a fuller understanding of fetal hemodynamics, measurement of the oxygen content of fetal blood would be a very helpful addition to flow quantification. With the development of fetal MR oximetry and blood oxygen level dependent imaging, a better understanding of oxygen handling by the fetal circulation may be available in the future [30,31,54]. We also lacked any long term neuro-developmental follow up in this group and believe further studies to examine the mechanisms and importance of fetal brain developmental delay in CHD should be a priority.

## Conclusions

PC CMR facilitates an understanding of the distribution of the fetal circulation in left-sided CHD and offers those providing care an additional tool for monitoring progress during fetal life at no additional risk to the mother or fetus [55]. The findings are generally in keeping with previous estimates of flow in the fetal circulation in CHD, but offer a more comprehensive assessment of blood flow distribution in this setting. By combining PC CMR with fetal organ volumetry and T2-weighted imaging, CMR allows investigation of the impact of fetal hemodynamics on fetal brain and lung development, and has the potential to contribute to the planning of fetal therapy and postnatal interventions. The identification of pulmonary lymphangectasia due to pulmonary venous hypertension in fetuses with HLHS with I/HRAS may be helpful to confirm the presence of fetal pulmonary vascular disease in this most malignant subgroup of fetuses with left-sided CHD. With this initial cohort, we have shown that CMR is feasible for studying late gestation fetuses with left-sided CHD and propose that, with larger numbers and long term follow up, we might be able to offer additional information regarding postnatal outcome.

## Abbreviations

AA: Aortic atresia; AAo: Ascending aorta; AS: Aortic stenosis; CHD: Congenital heart disease; CMR: Cardiovascular magnetic resonance; CoA: Aortic coarctation; CVO: Combined ventricular output; DA: Ductus arteriosus; DAo: Descending aorta; FO: Foramen ovale; HLHS: Hypoplastic left heart syndrome; I/HRAS: Intact or highly restrictive atrial septum; LA: Left atrium; LV: Left ventricle; CMR: Cardiovascular magnetic resonance imaging; MPA: Main pulmonary artery; MS: Mitral stenosis; MV: Mitral valve; PBF: Pulmonary blood flow; PC: Phase contrast; PL: Pulmonary lymphangectasia; PV VTI: Pulmonary vein velocity time integral; PVO: Pulmonary vein obstruction; RA: Right atrium; RV: Right ventricle; SVC: Superior vena cava; UAS: Unrestrictive atrial septum; UA: Umbilical artery; UV: Umbilical vein.

## Competing interests

The authors declare that they have no competing interests.

## Authors’ contributions

BAH performed CMR examinations, measured the flows, and drafted the manuscript. JFPvA analysed the data, developed the fetal CMR technology, and drafted the manuscript. JF performed and analysed echocardiographic examinations. EJ performed echocardiographic examinations and provided guidance on fetal cardiology. LGW and SJY helped to develop the fetal CMR protocol. CKM provided guidance with analysis of the data and developed the CMR technique. MS designed the study, developed the fetal CMR protocol, performed CMR examinations, measured the flows, and drafted the manuscript. All authors read and approved the manuscript.
